# Sexual Dimorphism of the Human Tibia through Time: Insights into Shape Variation Using a Surface-Based Approach

**DOI:** 10.1371/journal.pone.0166461

**Published:** 2016-11-15

**Authors:** Hana Brzobohatá, Václav Krajíček, Zdeněk Horák, Jana Velemínská

**Affiliations:** 1 Department of Prehistorical Archaeology, Institute of Archaeology of the Academy of Sciences, Prague, Czech Republic; 2 Department of Software and Computer Science Education, Faculty of Mathematics and Physics, Charles University in Prague, Prague, Czech Republic; 3 Department of Anthropology and Human Genetics, Faculty of Science, Charles University in Prague, Prague, Czech Republic; 4 Laboratory of Biomechanics, Faculty of Mechanical Engineering, Czech Technical University, Prague, Czech Republic; Rensselaer Polytechnic Institute, UNITED STATES

## Abstract

In this paper we present a three-dimensional (3D) morphometrical assessment of human tibia sexual dimorphism based on whole bone digital representation. To detect shape–size and shape differences between sexes, we used geometric morphometric tools and colour-coded surface deviation maps. The surface-based methodology enabled analysis of sexually dimorphic features throughout the shaft and articular ends of the tibia. The overall study dataset consisted of 183 3D models of adult tibiae from three Czech population subsets, dating to the early medieval (9–10^th^ century) (N = 65), early 20^th^ century (N = 61) and 21^st^-century (N = 57). The time gap between the chronologically most distant and contemporary datasets was more than 1200 years. The results showed that, in all three datasets, sexual dimorphism was pronounced. There were some sex-dimorphic characteristics common to all three samples, such as tuberosity protrusion, anteriorly bowed shaft and relatively larger articular ends in males. Diachronic comparisons also revealed substantial shape variation related to the most dimorphic area. Male/female distinctions showed a consistent temporal trend regarding the location of dimorphic areas (shifting distally with time), while the maximal deviation between male and female digitized surfaces fluctuated and reached the lowest level in the 21^st^-century sample. Sex determination on a whole-surface basis yielded the lowest return of correct sex assignment in the 20^th^-century group, which represented the lowest socioeconomic status. The temporal variation could be attributed to changes in living conditions, the decreasing lower limb loading/labour division in the last 12 centuries having the greatest effect. Overall, the results showed that a surface-based approach is successful for analysing complex long bone geometry.

## Introduction

The shape of the human lower limb bone has been shown to respond to environmental conditions and hormonal factors, together with body size/proportions and—foremost—physical load [1]. The issue of human tibia design has been studied extensively, but research has predominantly dealt only with shape variation of the shaft [2–5]. A few studies have focused on the shape of the articular ends [6–9] and even fewer have considered the whole tibial external geometry [10,11].

Traditional morphometric studies of human long bones based on manual measurements of angles/distances provided with only sparse anatomical information. Newer geometric morphometric approaches typically employ landmarks or semi-landmarks. Such methods can communicate complex shape changes much more effectively, and provide information on morphological changes in their immediate anatomical context. In the tibia, landmarks can be defined reliably only at its proximal and distal extremities, and shape differences are visualized as relative shifts of landmark positions in starting and target shapes [6,9,12]. A somewhat larger part of the shape can be captured by semi-landmark-based approaches [11] but still relatively large portions of the bone cannot be examined and some information is lost to analysis. To overcome such limitations for bone shape research, vertebrate palaeontologists have tested grids of points fitted to 3D models in carnivoran calcanei [13], aligned digitized surfaces using correspondence points in primate calcanei [14], and applied eigensurface analysis in avian humeri [15].

A whole-surface approach is gaining popularity for exploring the complex geometric shape variations of anatomical structures. Hutton et al. [16] have introduced an interpolation method using dense surface models. To date, this has been used mainly to analyse 3D facial morphology by establishing a correspondence of thousands of points across each 3D facial image. This approach has demonstrated great value in both biological and medical research. It has proven successful in delineating brain and facial morphology in genetic disorders [17], face growth trajectories [16], facial sexual dimorphic traits [18], shape variability of palatal surface in cleft patients [19] and facial asymmetry [20]. While whole-surface algorithms were developed originally for human faces, it can be applied to any type of surface data where there is a natural correspondence. Regarding full-surface long bone data, they have been studied within comparative anatomy [21], biomedicine [22–24] and forensic anthropology (to match paired elements) [25].

Because of its robustness and post-depositional resistance, the human tibia is an important skeletal element for identifying sex in both archaeological and forensic contexts [26–29]. Sex-related differences in the proximal tibia have also been studied extensively by orthopaedic surgeons. Within the orthopaedic community, the clinical relevance of sexual dimorphic traits is discussed mainly in connection with knee implant design [30]. However, the overwhelming majority of the literature devoted to sexing the human adult tibia deals only with conventional metric procedures. More recently, we have studied tibial sexual dimorphism (SD) from a geometric morphometric perspective; using landmark representation of shape to assess variation among two modern samples helped us describe significant sex-related differences in tibial extremities. However, sex-based divergence varied between the datasets analysed, raising issues of population specificity and diachronic change regarding these highly dimorphic structures [7].

The samples used for the current study varied in terms of chronology, living conditions and physical activity. The degree of SD is considered to provide an indication of the general health status and environmental stress, with weaker manifestation in populations exposed to poorer living conditions, and vice versa: stronger SD is expressed in populations experiencing better living conditions and health status [31,32]. Living conditions act through biological factors (nutrition and infections), both of which can affect adult body size [33]. Recent variations in body size SD probably reflect differences in subsistence strategy, diet and possibly sex-related buffering against the environment. In earlier time periods, these factors increased SD, because male growth is more sensitive to environmental agents [34]. During the last two centuries in particular, adult height and weight have been increasing in Europeans [35], inevitably impacting weight-bearing elements such as the tibia [36].

The chronologically oldest (9–10^th^-century) tibia dataset for this study originated from an early medieval agglomeration in Mikulčice (Czech Republic), both from the castle and its suburban area. The location of the burial ground and grave goods indicate a higher social status for the individuals buried in the castle (nobles, clergy and military escort), with a considerable proportion of people connected directly with the princely palace. In the adjacent sub-castle, craftsmen and other people involved in the functioning of the stronghold may have been based. On the whole, Mikulčice inhabitants lived in favourable living conditions, validated by studies of skull shape asymmetry [37]. In contrast, the second sample (the 20^th^-century dataset), derived from the Pachner collection, largely represented people from lower socioeconomic groups with adverse and stressful living conditions [38]. Their poor living standards, nutritional hardships and high environmental stress during development were expressed as high values of fluctuating asymmetry in the studies of Kujanová et al. [39] and Bigoni et al. [37]. For the chronologically youngest group (the 21^st^-century dataset), details of the socioeconomic context were not known. Despite this, they could be described as having experienced the best quality of life regarding nutrition, individual health and degree of stress from different origins.

Over the time period studied, the increasing industrialization in Europe was probably coupled with reduced physical strain/mobility during the populations’ daily routines. An exploration into biomechanical research has shown that the biomechanical signature of mobility is more marked in the diaphysis of the tibia relative to the femur [40]. Although loading history can only be roughly estimated in (pre)historic groups, we assumed that the amount of physical activity gradually declined during the studied time period (9^th^–21^st^-century). Previous studies have provided evidence for a decline in SD of tibial mid-shaft robusticity from pre-agricultural to agricultural and then industrial/modern societies [2,3], driven primarily by declining male anteroposterior strengthening of this element [2]. Macintosh et al. [41] have also documented a gradual decline in mobility that is consistent across Central Europe from 5300 cal BC to 850 AD. The diachronic change in lower limb bone morphology they showed was pronounced in both sexes but particularly in male tibiae. Across a wider time period, research by Berner et al. [42] has revealed a trend of declining SD in tibial bending strength from the Mesolithic to the Neolithic followed by a fluctuation in later periods. According to Ruff et al. [43], a decline in tibial bending strength continued through the Iron/Roman period (2000 BP) with no subsequent directional change until the 20^th^ century. Based on this comprehensive study, surprisingly tibial cross-sectional geometry has not mirrored increasing industrialization in Europe through the later Holocene in either males or in females.

As a follow-up to previous landmark-based studies, we extended the shape analyses to the whole tibial surface by adopting the idea of surface-based registration proposed by Krajíček [44]. This method enables the preservation of anatomical correspondence across the bony surfaces, and was applied to a series of Central European tibiae dating from the early medieval period to the present day. Analysing whole bone digital representations, our study aimed to characterize tibial sex-dimorphic characteristics and their potential temporal change across the last 12 centuries. We addressed three specific research questions.

First, whether the proposed method, engaging with whole-surface data, increases the accuracy of sex determination from the tibia. Initially we aimed to test its potential applicability for bioarchaeological and forensic investigations.

Second, to what extent, if any, does allometric scaling influence the shape of the tibia. Given the large size range within human tibiae and its weight-bearing function, allometry is likely to play some role in determining the morphology of its shaft and extremities.

Third, does the manifestation of SD in tibial shape vary across the time period studied? We intended to document not only male and female characteristic features with their discriminatory power but also potential differences in the pattern and degree of SD between one preindustrial and two modern datasets. Following on from previous biomechanical research [43], we assumed that functionally related SD would not strongly distort our findings and possible changes in degree of SD would to a greater extent be environment-related. Given these facts, it was hypothesized that sexually dimorphic traits would be most marked (because of the quality of life) in the contemporary Czech population and expressed least in the 20^th^-century dataset.

## Materials and Methods

The study was based on 3D models from individuals of Central European ancestry (Czech Republic) dating to three different time periods. The three datasets yielded 183 (65, 61 and 57) models of tibiae. Only adult bones, where epiphyses were completely fused, were scanned, either optically or with computed tomography (CT). Those individuals displaying any type of pathology or anatomical deformation that could affect the analysis were excluded.

### Skeletal datasets

The oldest group (an archaeological sample from the 9–10^th^ century AD) consisted of 65 left-side tibiae of adults aged from 20 to 60 years, unearthed from the early medieval burial area in Mikulčice (Czech Republic) (Table 1). Only well-preserved tibiae without pathology from adult individuals of known sex and age at death were included. The basic palaeodemographic characteristics, sex and age at death, were taken from data in the archive of the Department of Anthropology of the National Museum, Prague. Sexual classification was verified using Brůžek’s visual method [45]. Age at death was estimated according to a combination of methods from Buikstra and Ubelaker [46] and Schmitt et al. [47]. All the human skeletal remains from the locality of Mikulčice are stored at the Department of Anthropology of the National Museum, Prague.

**Table 1:** Characteristics of the datasets by age and sex. (N—number of individuals; M—males; F—females)

Dataset	All age groups			20–40 years			40–60 years			over 60 years		
	N	M	F	N	M	F	N	M	F	N	M	F
**9-10**^**th**^ **century**	65	35	30	28	11	17	37	24	13	0	0	0
**20**^**th**^ **century**	61	31	30	11	1	10	30	18	12	20	12	8
**21**^**st**^ **century**	57	30	27	3	2	1	17	14	3	37	14	23

The second dataset (the early 20^th^-century sample) consisted of 61 left-side non-pathological tibiae from the documented skeletal Pachner collection (31 tibiae from men ranging in age from 35 to 87 years, and 30 tibiae from women ranging in age from 20 to 72 years). Demographic data were acquired from autopsy records. This skeletal collection is curated at The Institute of Anatomy, First Faculty of Medicine, Charles University in Prague, Prague.

### Modern dataset

The most recent group (the 21^st^-century dataset) comprised 3D models of left tibiae from 57 living individuals. The bony surfaces were extracted from clinical anonymized CT scan sequences of adult individuals who had undergone CT angiography (during 2010–2013) (30 men ranging in age from 31 to 68 years and 27 women ranging in age from 33 to 91 years) (Table 1). As above, we included only normal, non-pathological bone without any indication of injury or advanced degenerative and senescence characteristics in the analysis.

### Scanning procedures

3D polygonal meshes were obtained via optical scanning of the skeletal datasets and generated from the CT scans of the modern-day dataset. To digitize the dry bones, a smartSCAN 3D-HE scanner (Breuckmann, GmbH, Meersburg, Germany) was used. This topometric system works on the basis of fringe projection on the object and recording of the modified patterns with two digital cameras with a resolution of 1.4 Mpix. Based on the system configuration [field of view M-600, 480 mm × 360 mm] used, the resolution in both the x and y axes was 360 μm. The resulting datasets, imaging the bony surface in four different positions, were processed and merged using OPTOCAT software (Breuckmann, GmbH, Meersburg, Germany) to make the final data object. Once the bony surfaces were scanned and meshes created, they were exported and saved in the.obj format used to store 3D graphical data of objects described by a series of polygons.

Another 57 surface models of modern-day tibiae were created using reconstruction methods employing virtual 3D modelling from the Digital Imaging and Communications in Medicine (DICOM) image sequence of CT outputs. The raw data were acquired by scanning the lower limbs using a Siemens Definition AS+ CT system (Siemens, Erlangen, Germany). The slice increment was set at 0.5 mm, with an X-ray tube adjustment of 120 kV and 51.6 mAs. The field of view was defined by a matrix of 512 × 512 pixels in 12-bit grey-scale levels. The pixel size was 0.977 mm. These adjustments correspond to the standard settings for angiography examinations.

A 3D models of the reconstructed tibial bony surfaces were created from a series of lower limb CT scans using the specialized software Mimics (Materialise, Leuven, Belgium). All areas of the CT scan with a specified range of grey values (GV) corresponding to bone tissue were recognized and manually thresholded in the first segmentation step. The 3D geometric models were then built according to a semi-automatically generated mask, which defined the boundaries of the osseous structure precisely and covered them with a polygonal mesh surface (.obj format). The appropriateness of including both scanned and tomographic data has been justified previously [48,49].

### Morphological comparisons

Initially, each tibial surface was represented by a polygonal mesh consisting of 280 000–414 000 faces. To ensure proper comparison between models, we down-sampled each mesh to a consistent number of 25 000 faces using the Quadratic edge collapse decimation tool in the freely available MeshLab software (v. 1.3.4) [50]. Like other algorithms used for morphological correspondence, our approach requires marking several landmarks (anchoring points) on digitized surfaces. Thirteen landmarks for the proximal and eight landmarks for the distal tibia were used based on those of Brzobohatá et al. [6], and were labelled manually using Morphome3cs software (v. 2.0) [51]. Further details of the landmark digitization procedure and intra-observer error have been reported previously [6] (S1 Table; S1 Fig).

Decimated meshes were then analysed on a whole-surface basis using dense correspondence modelling. For dense correspondence construction, surface registration of the sample was performed. For group registration, we used template-based registration fitting individual meshes to a preselected base mesh template. In the first step, the meshes were rigidly aligned using landmarks. If shape–size is to be analysed, this step preserves the size of the individuals, otherwise shapes are all scaled to the mean centroid size (CS) derived from the landmarks. Then, the free form deformation function was optimized in order to decrease surface deviation as well as the corresponding landmark distances [52]. Correspondences were found between vertices of the base mesh and nearest neighbour points on a particular fitted mesh. These correspondences were unwrapped to obtain topologically equivalent data for each mesh for point distribution model creation or mean mesh construction.

High-dimension principal component analysis (PCA) was then applied to the correspondences to extract principal variations in shape or shape–size. Scores in these variations were considered as shape variables. The number of statistically significant shape variables was determined using a broken-stick criterion [53]. A statistical atlas of shape (mean shape and a linear combination of eigensurfaces) was created [22,23]. Statistically significant differences between two groups of shapes (e.g. sexes) were determined by two-sample Hotelling’s T2 test or its permutation variant if normality could not be guaranteed on the shape variables [54]. In all cases significance was accepted as P < 0.05.

The discrimination ability of shape or shape–size was determined using the cross-validation (C-V) success rate of the linear discriminant analysis (LDA) classifier. Other classification methods (support vector machines, quadratic discriminant analysis) were also attempted, without better results. Classifier shape-space partitioning geometry was used to determine the linear model to generate extreme shapes by sex, and linear regression of CS with shape variables was used to create a linear model to generate the average shape by size. For visualization of mean differences or modes of shape variation, colour maps were used to show the amount of deviation of corresponding points or the impact of mode of variation on shape. All statistical procedures were performed with Morphome3cs (v. 2.0) [51] and R statistic software (v. 3.2.2) [55].

## Results

### Surface-based sex determination from the tibia

The first set of analyses assessed the significance of sex-based differences between datasets using both shape–size and shape-only variables. The permutation tests revealed a statistically significant separation between males and females from the same time period and also in pooled data irrespective of their chronology (Table 2). Likewise, it returned significant results between all the chronologically distinct male and female groups (S2 Table).

**Table 2:** Summary of p-values and cross-validated LDA scores indicating bones accurately classified based on sex. (N—number of individuals; M—males; F—females; bold—the statistical significance of group differences)

Dataset	N	M	F	Correctly classified with C-V (%) (shape-size)	P-value (shape-size)	Correctly classified with C-V (%) (shape)	P-value (shape)
**9-10**^**th**^ **century**	65	35	30	84.85	**<0.0001**	66.67	**0,0002**
**20**^**th**^ **century**	61	31	30	85.25	**<0.0001**	60.66	**0,0221**
**21**^**st**^ **century**	57	30	27	76.79	**<0.0001**	66.07	**0,0354**
**Pooled**	183	96	87	83.61	**<0.0001**	71.58	**<0.0001**

**Fig 1:**
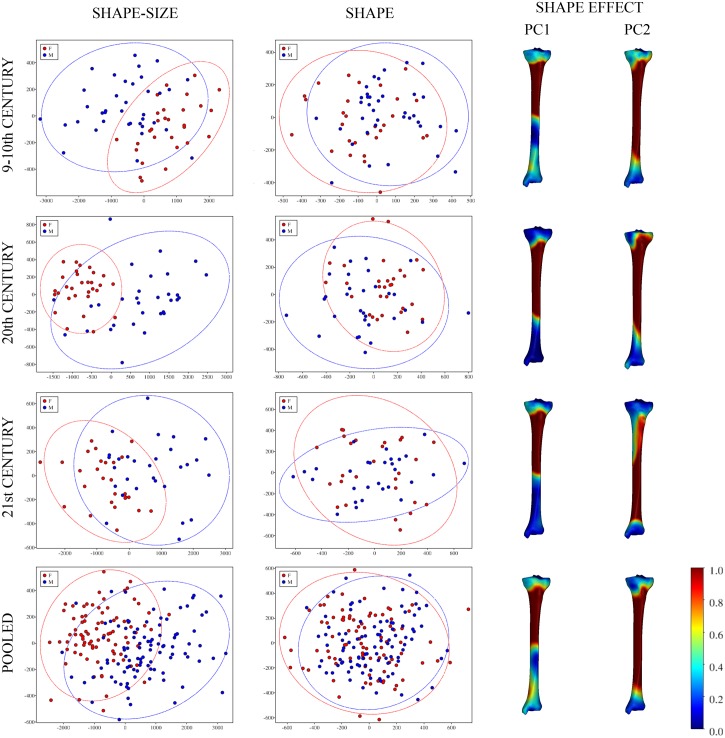
Scatter plots of PC1 (x-axis) vs. PC2 (y-axis) based on shape–size and shape variables. The left column represents shape–size bivariate plots, the middle column shape plots; groups of males (blue points) and females (in red) are delineated with 95% confidence ellipses. Colour-coded maps (right column) show the domains of tibial morphology most affected by particular PCs. Actual values represent deviation in mm caused by PC scaled by 10^4^.

PCA performed on shape variables showed that there was a substantial overlap between males and females in all the datasets studied. As expected, more pronounced distinctions between the groups were observed in PCA plots showing shape–size variation as was illustrated using the first two principal components (PC) (Fig 1).

As can be seen from same figure, PC1 (which comprised 19.45%–28.83% of the total shape variation) always reflected shape changes located on the upper half of the tibial shaft (the anterior projection of the anterior crest and tuberosity elevation). In contrast, the effect of PC2 (comprising 16.97%–19.09% of the total shape variation) varied visibly through time (Fig 1), as did the remaining PCs (not displayed).

When examining the accuracy of sex determination, the results of LDA with C-V using the PC scores from the shape–size space ranged from 76.79% to 85.25%. Excluding size had a major impact on the results. The values reflected the incomplete separation of sex groups obvious in scatter plots (Fig 1), with a correct classification level of only 60.66%–66.67%. When the three chronological datasets were pooled, the success rate increased slightly to 71.58% (Table 2).

**Fig 2:**
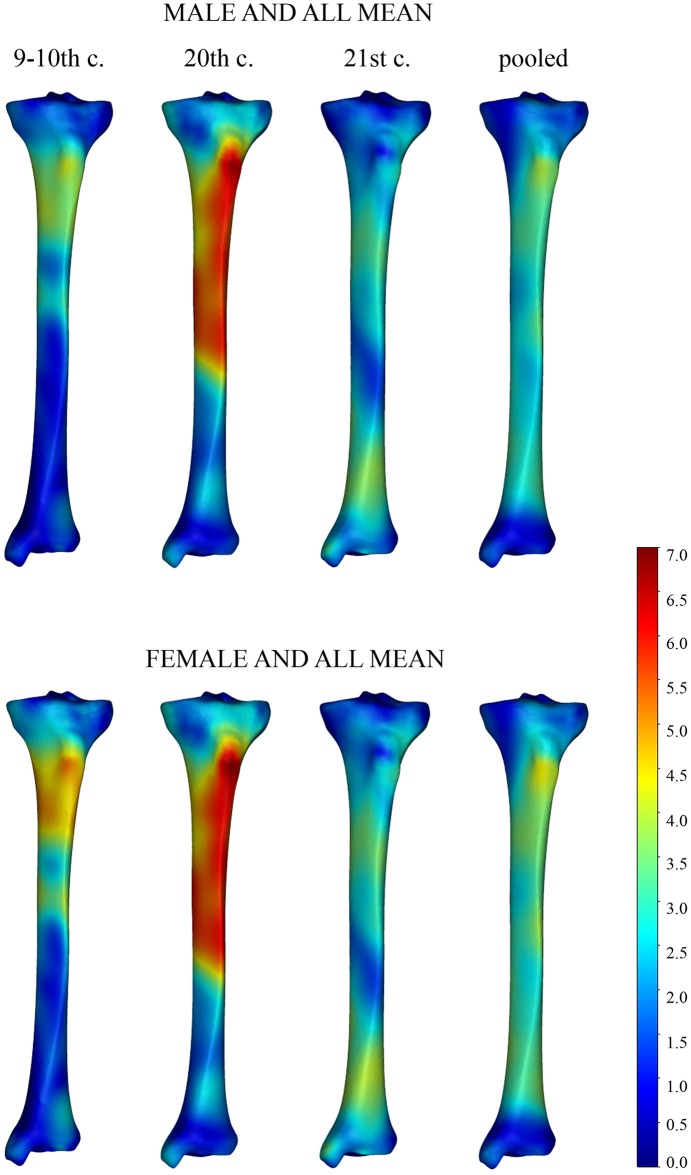
Frontal view of temporally changing sex-based tibial differences depicted by surface deviation maps. Deviations of the mean male (first row) and mean female (second row) meshes from the mean sample mesh are depicted by a colour scale (in mm). Blue tones indicate regions of minor mesh deviations, red-yellow tones illustrate the most dimorphic areas. The chronological age of the samples decreases from left to right.

**Fig 3:**
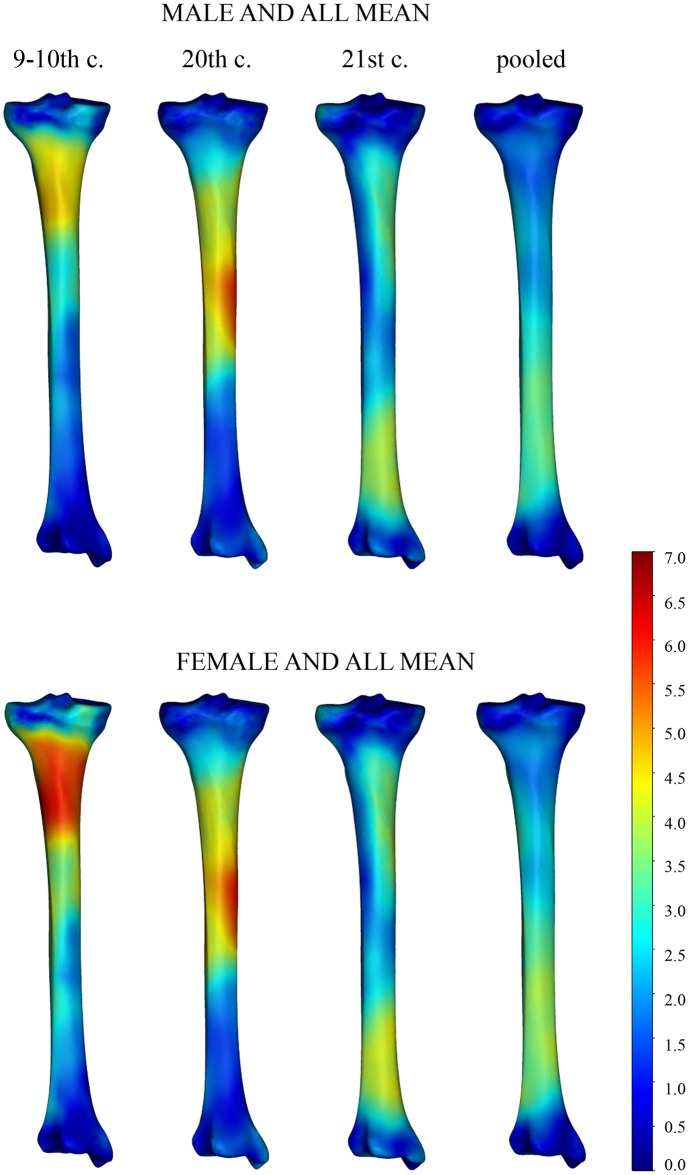
Dorsal view of temporally changing sex-based tibial differences depicted by a colour scale. (For legend see Fig 2.)

Further analyses related only to tibial shape variation, i.e. to the shape variables after removing all non-shape information. To identify the areas of tibial morphology that distinguished males from females for a given time period, a registration process was applied to the mean shapes derived from the sex groups. After registration of mean shapes, we quantified the surface-to-surface deviations for the whole bones. The impact of the individual´s sex on tibial shape was visualized with colour maps, where blue represented the minimal deviation and yellow/red indicated the largest deviation between the mean meshes of given sex and date. The most divergent regions differed up to 4.5 mm in the 21^st^-century dataset and 7 mm in the 9-10^th^-and the 20^th^-century datasets. In general, male and female tibiae could be differentiated based on the protrusion of tibial tuberosity and shape of the shaft (Figs 2 and 3). Further assessment of the colour maps is reported below, in Temporal trends.

**Fig 4:**
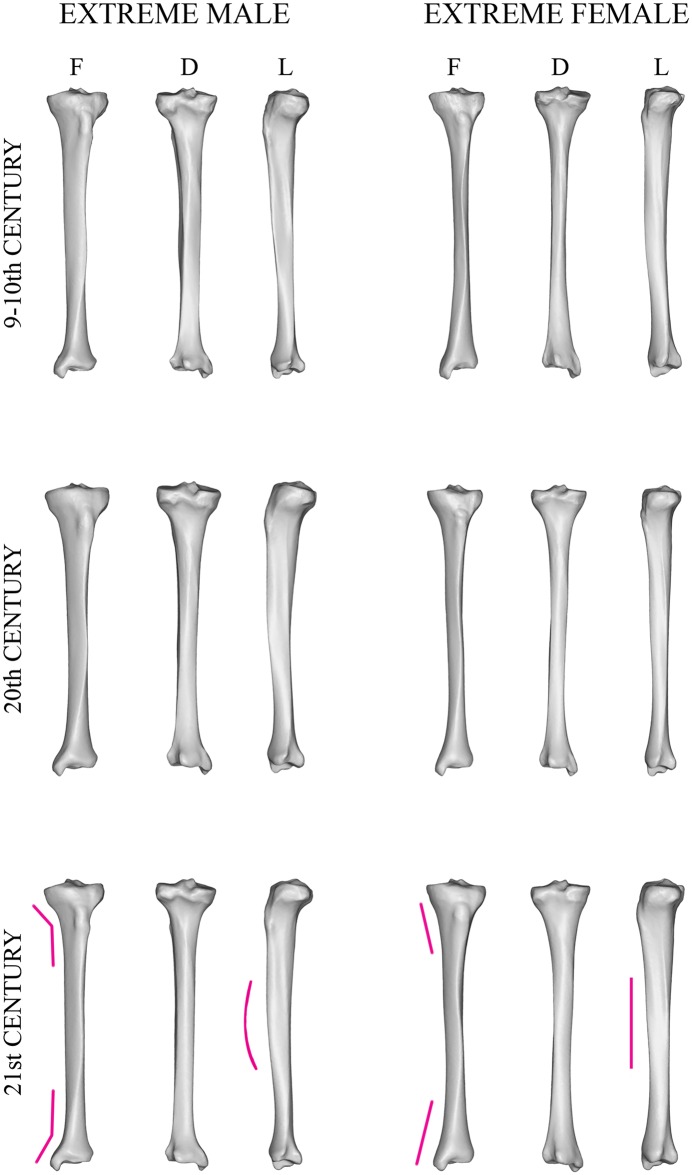
Extreme male and female tibiae. Extreme tibial shapes, which are generated at points in shape space on a line orthogonal to the plane separating sexes and passing through mean and which are situated in a certain distance (30 times sigma) from the mean on both sides. Males are shown on the left, extreme female shapes on the right, both in frontal (F), dorsal (D) and lateral (L) views. The chronological age of the samples decreases from the top to the bottom; purple lines point to the most sexually dimorphic areas (exemplified using the 21^st^-century dataset).

The main features separating males from females were also illustrated by extreme shapes. In males, the tibiae were more robust, with relatively larger extremities and a shaft that bowed anteriorly, apparent in side view. The male tibial tuberosity projected more anteriorly than the female tuberosity. The transition between shaft and articular ends was abrupt, which gave the lower border of the condyles a sharper edge in male compared with female bones. The frontal view showed a minor distinction between the datasets regarding the sigmoidal curvature of anterior crest: in the two older datasets the anterior crest followed a typical laterally concave/convex course down the front of the shaft in both sexes, but in the 21^st^-century group this mediolateral curvature was more accentuated in females. In general, female tibiae were more gracile and slender, with relatively smaller and narrower condyles and malleoli and a straighter shaft when viewed from the side. Female bone shafts progressively widened to the condyles and malleoli, which created a smooth transition between the shaft and the extremities (Fig 4).

### Size effect

**Fig 5:**
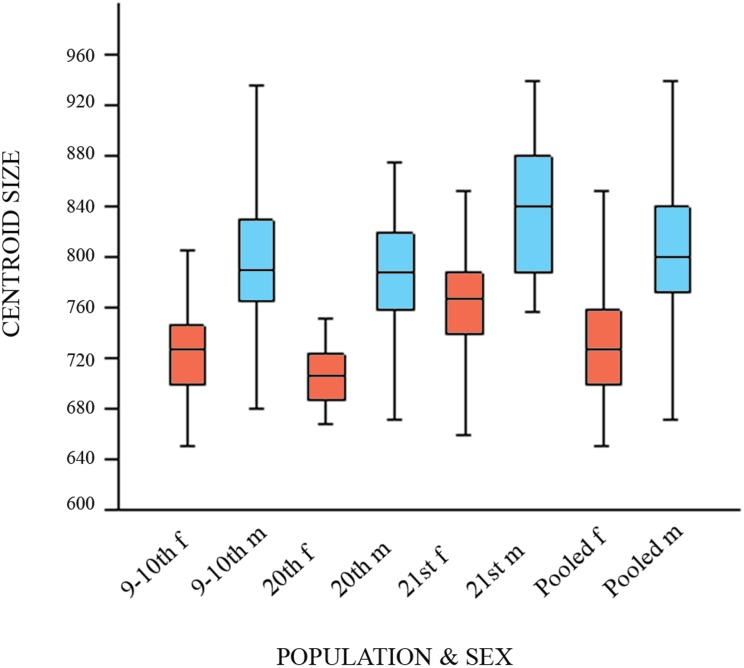
Variation of human tibia CS between chronologically diverse datasets. (9–10th m, f—9–10^th^-century dataset; 20th m and f—the 20^th^-century dataset; 21st m and f—the 21^st^-century dataset; f, females, displayed in red; m, males, in blue).

The variation in tibial size between chronologically diverse datasets is shown in Table 3 and Fig 5. Female CS (here used as a proxy for whole tibial size) decreased significantly in the first time span (from the 9-10^th^ to 20^th^ century) and markedly increased in the second period (from the 20^th^ to 21^st^ century). When we focused on male CS only, the 9–10^th^-century males did not differ significantly from the 20^th^-century males, and during the second time period the same trend was seen as in females (Fig 5 and Table 4).

**Table 3:** Average centroid size (avg. CS) by sex and dataset, and their relative differences.

	9–10^th^ century	20^th^ century	21^st^ century	Pooled
**Female avg. CS**	723.41	704.05	760.85	728.41
**Male avg. CS**	797.38	784.73	837.75	805.49
**Relative difference (%)**	10.23	11.41	10.11	10.58

**Table 4:** P-values of permutation t-test of centroid size (same mean). To test for differences between CS, permutation tests with 2000 replicates were performed. Results are reported with a significance level of P < 0.05, displayed in bold.

Dataset	Males		Females	
	9–10^th^ century	20^th^ century	9–10^th^ century	20^th^ century
**20**^**th**^ **century**	0.315		**0.0237**	
**21**^**st**^ **century**	**0.005**	**0.0003**	**0.0008**	**<0.0001**

**Fig 6:**
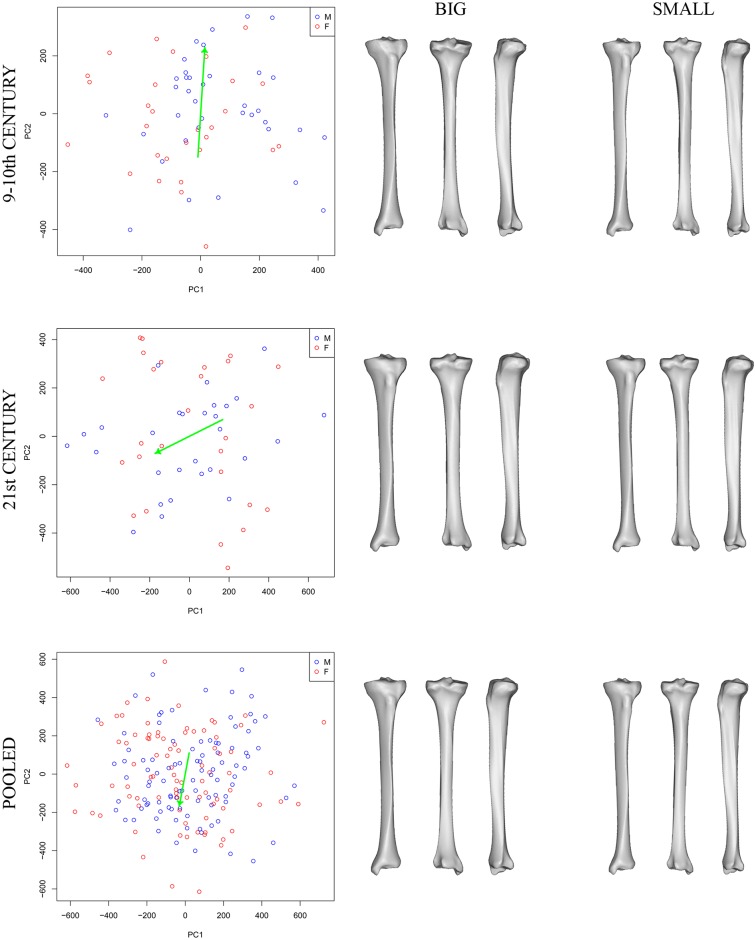
Scatterplots of the first two PCs of tibial shape visualizing static allometry. The green arrow represents static allometry, miniatures (right) visualize the shapes at the extremes of allometric variation.

To test whether the intra-population sex-based differences were associated with differences in bone size, we controlled for the potential impact of allometry. For each dataset and pooled data, linear regressions were performed on CS as an indicator of size, regressed against individual PCs. The results demonstrated that CS was significantly correlated with shape in all datasets with the exception of the 20^th^-century sample. In the 9–10^th^-century sample, allometry was closely aligned with PC2 and PC6, in the 21^st^-century group with PC1 and in pooled data with PC2 and PC3 (Table 5). Each time the allometric shape change essentially imitated the sex-based differences: these included a more anteriorly bowed shaft combined with relatively more robust extremities in larger bones, while smaller tibiae (analogous to female tibiae) were characterized by straighter shaft and more slender articular ends (Fig 6).

**Table 5:** Statistical values of the linear regression of shape vs. CS for all groups. (R^2^ –multiple R squared; significant p-values displayed in bold).

Dataset	9-10^th^ century			20^th^ century			21^st^ century			Pooled		
Component	Explained variance	R^2^	P-value	Explained variance	R^2^	P-value	Explained variance	R^2^	P-value	Explained variance	R^2^	P-value
**PC1**	21.29	0.001	0.838	28.83	0.014	0.361	23.54	0.071	**0.045**	19.4550	0.001	0.547
**PC2**	19.19	0.187	**0.0002**	16.97	0.003	0.642	18.38	0.015	0.361	17.0789	0.065	**0.0004**
**PC3**	12.39	0.023	0.219	12.19	0.042	0.110	11.64	0.053	0.087	14.6676	0.034	**0.012**
**PC4**	7.16	0.004	0.592	8.49	0.023	0.235	9.26	<0.001	0.874	8.77	<0.001	0.791
**PC5**	5.58	0.007	0.502	5.24	0.041	0.113	5.30	0.024	0.249	5.78	0.008	0.215
**PC6**	3.11	0.163	**0.007**							3.00	<0.001	0.986

### Temporal trends

The co-registered meshes and extreme shapes provided primary data on long-term changes in SD pattern and degree. All three datasets had three sex-dimorphic features in common. Proximally, the elevation of tibial tuberosity protruded more anteriorly in male tibiae; and males had relatively larger and wider articular ends and anteriorly bowed shafts than females (Fig 4).

Apart from these features, which remained more or less constant through time, we detected characteristics unique to each of the datasets. The most typical trend seen in the colour maps was a shift in the dimorphic area (displayed in red or yellow tones) over time. Early medieval (9–10^th^ century) tibiae differed most in the upper quarter of the shaft, i.e. at the level of the tibial tuberosity and below. Along the remaining length, the bone surfaces roughly coincided. For the 20^th^-century tibiae, the most dimorphic area was similarly localized, but it expanded more distally to the whole upper half of the shaft. Finally, in the 21^st^-century dataset, this region was expanded more distally, but was broken by a zone of relatively smaller differences below the middle of the shaft. The main differences were localized around the upper third of the shaft and at the shaft thinning above the distal articular end. For the pooled data, the biggest computed distances were proximally around the tibial tuberosity and distally above the ankle. In all three datasets, there was pronounced diminishment of mesh deviation at both articular ends, delineating areas where sex had the least impact (Figs 2 and 3).

## Discussion

This study shows sex-specific and temporal variation in human tibia morphology. We tested the potential applicability of surface-based methods to sex determination of the tibia and whether additional surface information can augment the predictive ability of previous sex classification methods. In addition to documenting male and female features within chronologically distinct datasets, we looked at the potential allometric effect and temporal changes of the SD pattern over time.

Currently, sex classification of long bones is based mainly on traditional morphometric tools. For the tibia, the accuracy in sex determination varies between measurements and studies, but generally the most reliable indicator of sex is the proximal breadth. A combination of several tibial parameters usually provides a high degree of accuracy, from 80% to 98% (without C-V) [26–29].

Since geometric morphometric has been established as a discipline, new techniques have emerged to differentiate between male and female long bones [7,56]. Using landmark methodology and proximal tibial data, sex has been diagnosed with a high degree of accuracy (87.5%–91.80% after C-V) when size and shape are combined [7]. Even though sex determination based on whole 3D models is often accurate enough [57], the surface-based techniques applied in this study (with success rates of 76.79%–85.25% for shape–size and 60.66%–71.58% for shape data) proved to be less successful than landmark methods. The accuracy of prediction achieved a level considered acceptable only in general osteological, but not forensic, contexts [58]. For the pooled dataset, an accuracy of 71.58% was attained, which indicates that the low accuracy could have been the result of the small dataset sizes, and thus the SD became more distinct when the data from the three datasets were combined.

The CS values that we calculated from landmark configurations covering proximal and distal tibia comprised both the tibial length (reflecting body height) and the robustness of its articular ends (which is good predictor of body weight) [59,60]. Contrary to the hypothesis that male growth is less buffered against environmental stress [34], our results showed that female tibiae reflected changes in living conditions to a greater extent than males, with a significant decrease in CS from the earliest time periods (from the 9–10^th^ to the 20^th^ century) and subsequently a marked increase from the 20^th^ to the 21^st^ century. Regardless of the poor living standards of the 20^th^-century dataset, male CS did not change during the earliest time period and increased during the second time period, identical to the females.

An allometric effect was found in the chronologically oldest, youngest and pooled datasets, while in the 20^th^-century population size was not a critical determinant of the whole tibial shape. Biomechanical research has shown that the morphology of lower limb bone shafts is influenced by the combined effect of body mass and physical activity [61]. The early medieval dataset probably experienced the highest physical load. The allometry in this group suggests that tibiae may have responded to increased bone/body size when combined with higher habitual levels of activity. In turn, the size-related shape change observed in the 21^st^-century dataset could have been linked to an increased body size (reflected in the CS increment). Features that are allometric consequences of bone size–increased shaft bowing and extremity robustness in larger tibiae—were in fact identical to the sex-based characteristics of male and female bones.

Although there was no improvement in sex classification rates when using whole polygonal meshes, our approach provided a more comprehensive analysis of sex-related shape variations in human tibia. Most features previously described in the literature that have been used to distinguish male from female tibiae have been strictly metric in nature and related predominantly to epiphyseal bone measurements [26–29]. Metric analyses suggest that, when these parameters are measured on male tibiae, higher absolute values are obtained; the results of this study support the conclusion that males have larger articular ends both absolutely and relatively. Placing greater emphasis on shape as opposed to size, we can compare our results only with the classic morphoscopic investigation of Hrdlička [62]. Apart from relatively larger male extremities, he noted persistence in the female tibia of more or less the infantile bone character [62]. In contrast to metric sexing techniques, our data show that maximally dimorphic regions (potentially the best discriminators) are actually located in non-articular bone regions. Despite being highly accurate for metric sexing, articular areas contribute least to overall sex-based variance and remaining bone structures could serve as more reliable sex estimates. In particular, the shape of the transition between the shaft and extremities, protrusion of the tibial tuberosity, and curvature of the anterior shaft are all tibial features located in the most resilient bone regions and are more likely to survive prolonged burial and subsequent excavation. Our findings may thus initiate further studies specifically targeted at the use of these features for classifying fragmentary bones. We could show that sex differences were particularly accentuated in the shaft and diminished towards the proximal and distal portions of the tibia, which are highly genetically canalised characteristics and functionally constrained by the neighbouring anatomical structures [61,63]. Moreover, closer examination of colour-coded maps within the temporally distinct datasets from the same geographic region revealed two important observations. First, that there was significant temporal variation regarding the pattern of SD, and second that the degree of SD also changed over time.

The two chronologically older datasets exhibited similarly located dimorphic areas and temporal changes within them were weaker. More striking changes occurred during the second time period, in less than a hundred years. Although the trend of dimorphic area expansion continued, mesh-to-mesh deviations were in fact reduced. Together this resulted in the modern-day manifestation of SD characterized by two moderately dimorphic areas spreading out over almost all the tibial shaft. These differences seen in the expression of SD between the three datasets may be the result of a combination of several factors.

Although the datasets we studied were geographically identical, some gene flow can be expected as a result of population movements over time. Adult long bone morphology is a complex product of genetic and environmental interactions [64,65] but major evolutionary features may be principally genetic and the remaining skeletal features are more or less environmentally modifiable [65]. Thus we assume that most of the observed temporal variation is derived from epigenetic influences such as climatic, dietary and behavioural factors [66].

Numerous findings indicate that climatic factors continue to be significant correlates of world-wide variation in human body size and shape [40,67]. For the study region and time periods, mean annual temperature oscillations have only been minor [68]. We therefore conclude that only a negligible (if any) part of the variation seen in inter-population differences in bone shape is related to climate.

One possible reason for the differences seen between the datasets could be bone remodelling as a result of aging or, more precisely, an imbalance in age cohorts [69]. Recent geometric morphometric studies exploring age changes in the proximal tibia have revealed that although age-dependent shape variation is apparent and significant, inter-population and sex-related shape differences are considerably more important [6,9]. In the tibial shaft, where we saw the most marked SD, cross-sectional geometry age changes take the form of continuous expansion during adulthood (for review see [70]). Ruff and Hayes [71] reported skeletal remodelling with age that was most pronounced in the mid-distal tibia. In contrast to preindustrial population data, where both sexes showed the same pattern of change with advancing age, in recent adult Americans changes in diaphyseal geometry appeared in men only [71]. On the basis of these findings we deduce that age could have moderately increased the degree of SD observable in our modern datasets.

The most probable explanations for the temporal SD changes we observed are the substantial changes in living conditions and physical load that have taken place during the time period covered by our samples. The degree of SD in human populations is influenced by stress, social role and labour division [72]. The severe decline in living standards for the 20^th^-century dataset was reflected in the summary of correct sex determination percentages based on shape. Surprisingly this revealed the highest discriminatory power based on shape–size data from the 20^th^ century. In contrast, when LDA was performed on shape variables alone, it resulted in the highest proportion of unsexed individuals within this dataset. Their predecessors and successors with higher socioeconomic standard yielded higher LDA success rates based on shape (Table 2). Thus, the commonly held assumption of stronger SD expression reflecting better living conditions [31,32] was confirmed only by using surface data after removing all non-shape information.

Temporally varying sex-dimorphic features in tibiae represented by colour-coded maps raise the question of whether the environmental stresses were hidden or not by changing biomechanical requirements. Differently located and pronounced dimorphic areas could be indicative of more strenuous and sex-distinct lower limb loading regimes in two older datasets. As a result of increasing mechanization, the contemporary Czechs can apparently be assigned the lowest physical activity and very little (if any) dimorphism in locomotor behaviour. Based on the variation seen in the colour-coded maps, SD was expressed more in the chronologically older datasets. Thus, we could not confirm the hypothesis that sexually dimorphic traits as a result of the quality of life would be most marked in the contemporary Czech dataset. It logically follows that the SD changes documented here could in large part be related to lifestyle differences, with different functional demands placed on lower limbs in the different time periods.

In summary, we have demonstrated that changes in both the degree and pattern of shape SD can occur in long bones over relatively short timescales, in some cases in less than one century. Our results thus corroborate earlier studies that refer to the population specificity of sexual dimorphism and endorse the use of up-to-date CT-derived data for forensic investigations [73]. Varying profiles of tibial shape SD can most probably be attributed to a combination of changing environmental conditions and an increasingly sedentary lifestyle over time.

## Conclusions

We have presented an efficient method for performing comprehensive shape exploration over entire digitized bony surfaces by applying dense correspondence modelling. The specific problems we have addressed are its potential applicability for sex determination, the assessment of the importance of allometry in determining human tibial shape and the detection of temporal changes in shape sexual dimorphism in human tibia.

Analysing shape SD, we deduced that using whole polygonal meshes does not improve sex-classification rates compared with traditional and landmark-based morphometrics. However, a surface-based approach is better at determining how much each part of the bone contributes to the total sex-related variance. Our results have also provided evidence for considerable temporal variation in tibia shape SD over a relatively short time period from the early medieval to present day. By investigating shape SD in chronologically distinct datasets, we have described sex-specific tibial features that have remained constant over time and also some significant distinctions regarding the location and expanse of the most dimorphic areas. When testing for size-related shape changes, size was found to play an important role in all datasets with the exception of the 20^th^-century group and male attributions with bone enlargement were highlighted. We conclude that sex-specific tibial morphologies in the study datasets were influenced by differential levels of environmental stress, with a major contribution from changing physical load.

The resulting visualizations are encouraging for further study of shape variation within landmark-depleted structures. We also conclude that the use of whole bone digital representations allows a better understanding of shape differences, with the potential for such data to be assessed for diverse factors affecting external bone morphology.

## Supporting Information

S1 Fig Digitized surface of human tibia with proximally and distally placed landmarks. The proximal articular end is presented in frontal (A), dorsal (B) and superior (C) views; the distal extremity is shown in lateral (D), medial (E) and inferior (F) views. Refer to S1 Table for the landmark descriptions employed.
(TIF)

S1 File Mean dataset, male and female meshes (shape-only data) in. obj format. (ZIP)

S1 Table List of landmark numbers, descriptions and types according to Bookstein (1991). (XLSX)

S2 Table P-values from permutation version of Hotelling’s *T*^2^ test by sex and population (based on shape-only variables). (XLSX)
