# Supplementary figures and images for: Sexual Dimorphism of the Human Tibia through Time: Insights into Shape Variation Using a Surface-Based Approach
Source: PLoS One. 2016 Nov 15;11(11):e0166461. doi: 10.1371/journal.pone.0166461 (PMC5112946; doi:10.1371/journal.pone.0166461)

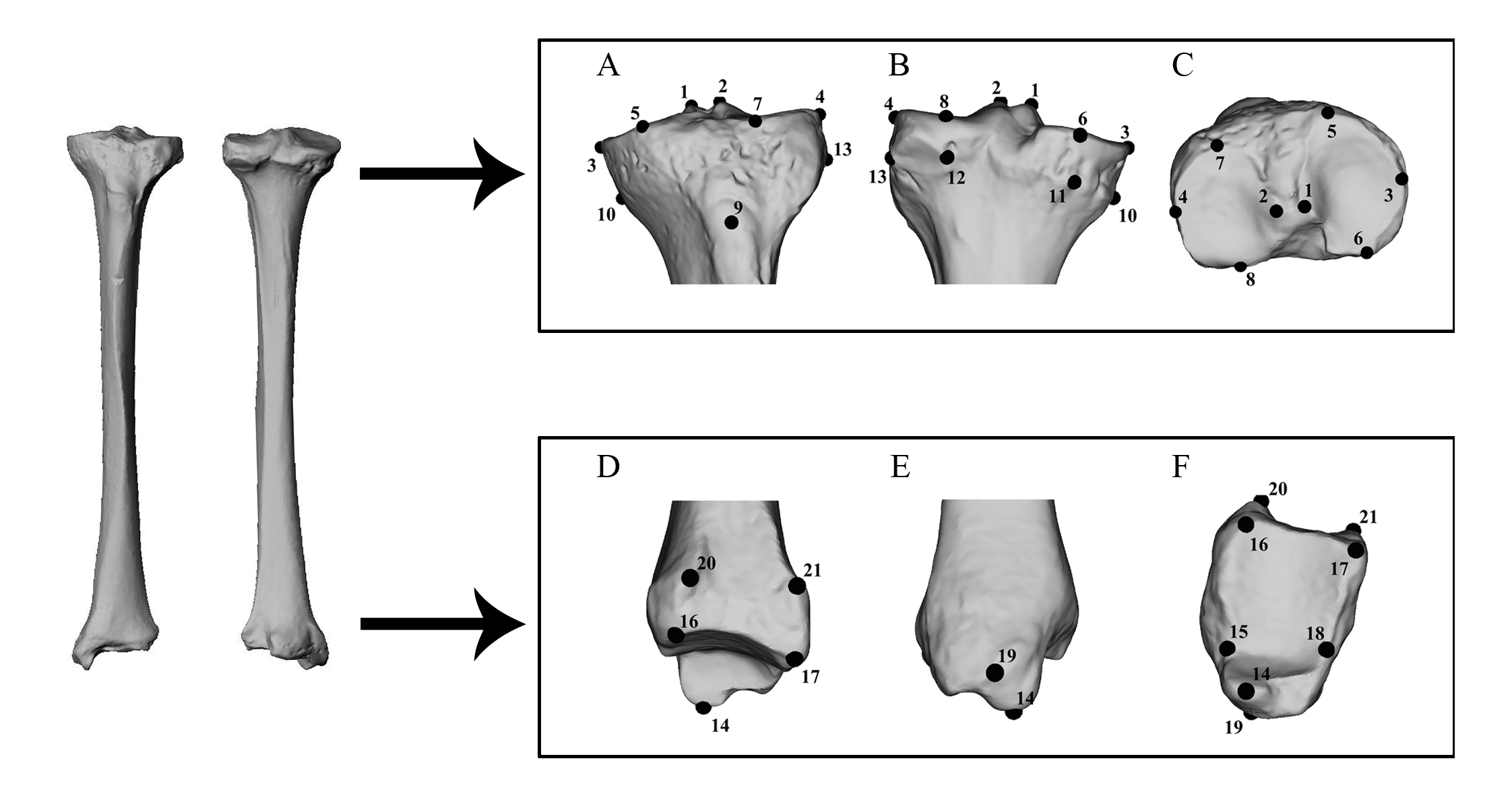

Supplement: S1 Fig — The proximal articular end is presented in frontal (A), dorsal (B) and superior (C) views; the distal extremity is shown in lateral (D), medial (E) and inferior (F) views. Refer to S1 Table for the landmark descriptions employed. (TIF) [file pone.0166461.s001.tif]
